# TSH reference values in the first trimester of gestation and correlation between maternal TSH and obstetric and neonatal outcomes: a prospective Brazilian study

**DOI:** 10.1590/2359-3997000000132

**Published:** 2015-01-01

**Authors:** Pedro Weslley Rosario, Marina Carvalho, Maria Regina Calsolari

**Affiliations:** 1 Santa Casa de Belo Horizonte Belo Horizonte MG Brasil Programa de Pós-graduação, Serviço de Endocrinologia, Santa Casa de Belo Horizonte, Belo Horizonte, MG, Brasil

**Keywords:** TSH, pregnant women, first trimester, reference values, obstetric and neonatal outcomes

## Abstract

**Objective:**

To define the normal range of TSH in the first trimester of gestation and to evaluate the correlation between maternal TSH and obstetric and neonatal outcomes.

**Subjects and methods:**

Prospective study. Women without known or clinically suspected thyroid disease and without risk factors for thyroid dysfunction, who became pregnant spontaneously and were initially evaluated up to week 12 of gestation, were included. Women with positive anti-thyroperoxidase antibodies, twin pregnancy, hyperemesis gravidarum, and trophoblastic disease were excluded.

**Results:**

In the 660 pregnant women, the mean, median, and 2.5^th^ and 97.5^th^ percentiles of TSH were 0.9, 0.96, 0.04 and 2.68 mIU/L, respectively. TSH was undetectable in 2%, < 0.5 mIU/L in 17.4%, > 2 mIU/L in 9.7%, > 2.5 mIU/L in 4.7%, and > 3 mIU/L in 1%. None of the women received levothyroxine or antithyroid drugs during pregnancy. In addition, there was no difference in obstetric or neonatal outcomes when women with TSH ≤ 0.1, between 0.1 and 2.5, and between 2.5 and 4 mIU/L were compared.

**Conclusion:**

In the population studied, the TSH value corresponding to the 97.5^th^ percentile was 2.68 mIU/L in the first trimester of gestation.

## INTRODUCTION

TSH is the most important test for the evaluation of thyroid function during pregnancy. Its measurement is essential for the diagnosis of primary thyroid dysfunction and is usually sufficient to exclude or suspect this condition. Supporting its importance, levothyroxine (L-T4) replacement therapy should be considered in pregnant women with elevated TSH, even in the presence of normal T4 concentrations and in the absence of anti-thyroperoxidase antibodies (TPOAb) (1-4), while L-T4 treatment is controversial in pregnant women with normal TSH, even in the presence of hypothyroxinemia or circulating TPOAb (1-4).

Serum TSH concentrations during pregnancy are not the same as those observed in nonpregnant women, and specific reference values are necessary. In this respect, knowledge of the normal range of TSH in the first trimester is the most important. First, greater differences in the concentrations of this hormone between pregnant and non-pregnant women are observed during this period (5). The extremely high circulating human chorionic gonadotropin (hCG) levels achieved in the first trimester activate the TSH receptor and thus directly stimulate the thyroid to produce more thyroid hormone which results in decreased TSH secretion. Second, before about 12 weeks of gestation, when the fetal thyroid gland becomes active, the mother is the sole source of thyroid hormones and maternal thyroid sufficiency might therefore be most important in the first trimester (6). Finally, there is agreement that, when indicated, screening for thyroid dysfunction should be conducted on the first prenatal visit (1-4), which generally occurs during the first months of pregnancy.

Rigorous selection of the sample is necessary to establish TSH reference values in pregnancy since, in addition to the habitually required exclusion criteria [known or clinically suspected thyroid disease, goiter, neck radiotherapy, personal history of autoimmune disease, family history of thyroid disease, drugs, and TPOAb (7,8)], there are potentially interfering obstetric conditions [twin pregnancy, hyperemesis gravidarum, trophoblastic disease (5)].

The objective of this prospective study was to define TSH reference values in the first trimester of gestation for a Brazilian population, and to correlate maternal TSH concentrations with obstetric and neonatal outcomes.

## SUBJECTS AND METHODS

### Prospective study

The population studied was from the metropolitan region of Belo Horizonte (Minas Gerais, Brazil). Pregnant women who underwent prenatal exams at a clinical analysis laboratory and who had become pregnant spontaneously were initially interviewed and examined (9). Serum samples were obtained from the women in the morning (at about 8 a.m.) after an 8- to 10-h fast (9). Seven hundred and forty-eight women who met the clinical criteria shown in Table 1 were first selected (9). Next, TPOAb and TSH were measured. TPOAb-positive pregnant women were excluded. Women with hyperemesis gravidarum, twin pregnancy or trophoblastic disease were also excluded (5).

**Table 1:** Inclusion criteria

Clinical criteria
Absence of thyroid disease, no current or previous treatment with antithyroid drugs or L-T4, no history of ^131^I therapy or thyroidectomy
No use of potentially interfering medications such as dopaminergic agonists or antagonists, neuroleptics, corticosteroids, estrogen, amiodarone, interferon, lithium, anticonvulsants, metformin, octreotide; or recent (in the past 8 weeks) exposure to iodinated contrast agents
No history of head and neck external radiotherapy
Absence of type 1 diabetes or other autoimmune diseases
No family history of thyroid disease
Absence of goiter or any palpable thyroid anomaly
Absence of ophthalmopathy
Complementary criteria: ≤ 12 weeks gestation and TPOAb negative

The final sample consisted of 660 women [a sample size larger than that required by the NACB (7)] ranging in age from 18 to 36 years (median 26.5 years), with a median gestation of 9 weeks, including 350 primigravidae.

Obstetric and neonatal outcomes were analyzed.

The study was approved by the local Research Ethics Committee (Santa Casa de Belo Horizonte).

TSH was measured with a chemiluminescent assay (Immulite 2000, Diagnostic Products Corporation, Los Angeles, CA), with reference values of 0.4-4 mIU/L, a functional sensitivity of 0.02 mIU/L, and intra- and interassay coefficients of variation < 7% for values ranging from 0.1 to 40 mIU/L. TPOAb were also measured with a chemiluminescent assay (Immulite 2000), with reference values of up to 35 kIU/L.

The reference limits of normal TSH were defined as follows: 1) 2.5^th^ and 97.5^th^ percentiles of the values obtained for the sample; 2) logarithmic transformation of the values obtained, calculation of the mean ± 1.96 SD of these values, and exponentiation to obtain the limits corresponding to the original scale (7). Fisher’s exact or c^2^ test were used to compare groups. P values < 0.05 were considered statistically significant.

## RESULTS

**Figure 1:**
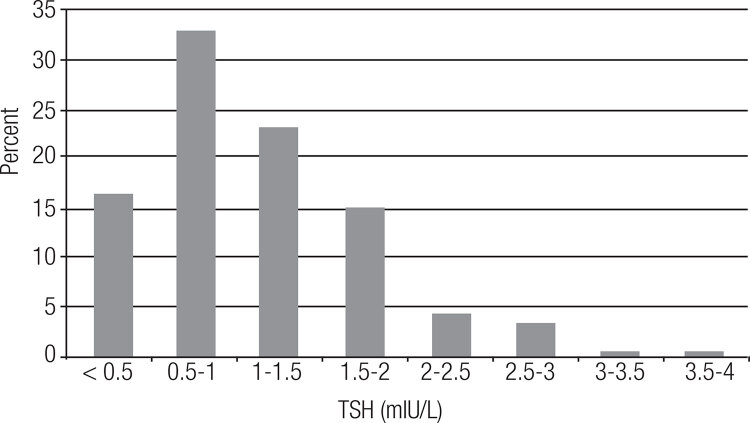
TSH distribution for the entire cohort (n = 660).

Figure 1 shows the distribution of TSH values in the sample. The mean, median, maximum, and 2.5^th^ and 97.5^th^ percentiles of the values obtained were 0.9, 0.96, 3.8, 0.04 and 2.68 mIU/L, respectively. TSH was undetectable in 2% of the pregnant women, < 0.5 mIU/L in 17.4%, > 2 mIU/L in 9.7%, > 2.5 mIU/L in 4.7%, and > 3 mIU/L in 1%. We found no difference in TSH values between primigravidae versus multigravidae (p = 0.51).

The upper-reference limit (mean + 1.96 SD) was estimated for the log-transformed data and then exponentiated back to the original scale. The resulting value (2.71 mIU/L) was very close to the 97.5^th ^percentile of the TSH values (2.68 mIU/L).

None of the pregnant women were treated with L-T4 or antithyroid drugs up to the end of pregnancy. Table 2 shows the obstetric and neonatal outcomes according to maternal TSH concentrations in the first trimester of gestation.

**Table 2:** Obstetric and neonatal outcomes according to maternal TSH concentrations in the first trimester of gestation

Obstetric and neonatal outcomes	Maternal TSH concentration		
	< 0.1 mIU/L (n = 33)^**a**^	0.1-2.5 mIU/L (n = 596)	2.5-4 mIU/L (n = 31)
Hypertensive disease of pregnancy^b^	1 (3%)	54 (9%)	3 (9.6%)
Placental abruption	0	2 (0.3%)	0
Delivery before 34 weeks of gestation	0	6 (1%)	0
Delivery before 37 weeks of gestation	1 (3%)	18 (3%)	1 (3.2%)
Fetal loss (after initial evaluation)	1 (3%)	18 (3%)	1 (3.2%)
Birth weight < 2,500 g	2 (6%)	35 (5.8%)	2 (6.4%)
Birth weight < 1,500 g	0	9 (1.5%)	0
Need for intensive therapy	0	12 (2%)	1 (3.2%)
Need for mechanical ventilation > 24 h	0	12 (2%)	1 (3.2%)
Necrotizing enterocolitis^c^	0	1 (0.16%)	0
Intraventricular hemorrhage grade 3 or 4	0	0	0
Major congenital malformations	0	6 (1%)	0
Neonatal death (< 28 days)	0	3 (0.5%)	0

^a ^All without thyroid disease and with TSH normalization after the first trimester.

^b ^Exlcuding women who were hypertensive before pregnancy.

^c ^With need for surgery.

## DISCUSSION

We first highlight some characteristics of the study. This is the largest Brazilian study evaluating TSH concentrations in healthy pregnant women, in which the number of participants was much higher than that recommended by the NACB (7), selection was performed rigorously (clinical and TPOAb), including potentially interfering obstetric conditions (5), and the samples were adequately obtained (after fasting and in the morning in all participants). Ultrasonography (US) was not performed because of the following reasons: (i) US is not required by the National Academy of Clinical Biochemistry [NACB (7)]; (ii) studies have shown no change in the reference limits of normal TSH when subjects with ultrasonographic anomalies were excluded from the initial sample (10-14), a finding also demonstrated in a previous study from our group (15); (iii) no consensus exists regarding the ultrasonographic findings to be considered (10-14), and (iv) many of the subjects with US suggestive of chronic thyroiditis without circulating TPOAb are identified based on a personal history of autoimmune disease, a family history of thyroid disease or presence of goiter, which were exclusion criteria in the present study.

Our study confirms that low TSH concentrations are relatively frequent in the first trimester of gestation. TSH < 0.5 mIU/L was observed in 17.4% of the pregnant women studied and ≤ 0.05 mIU/L in 3%. The main explanation for this finding is the action of HCG on the TSH receptor, which increases the secretion of thyroid hormones, with a consequent reduction in TSH. In fact, none of the women with undetectable TSH at the beginning of pregnancy had a history of thyroid disease, ophthalmopathy, goiter, palpable nodules, or TPOAb (exclusion criteria). The demonstration that low and even undetectable TSH in the presence of normal thyroid hormone concentrations in the first trimester of gestation is not associated with negative outcomes (16), and may even be a protective factor against some outcomes (16,17), supports the physiological nature of this finding. Therefore, treatment of pregnant women with antithyroid drugs should only be considered when undetectable TSH is accompanied by elevated levels of T4 and/or T3 and in the presence of underlying thyroid disease (most frequently Graves’ disease).

The upper limit of normal for TSH is the most important parameter in clinical practice for both adjustment of the L-T4 dose in patients receiving hormone replacement therapy and the indication of this therapy (1-4). In contrast to low TSH, which alone is not an indication for treatment, in pregnant women with elevated TSH L-T4 replacement therapy is indicated even in the presence of normal T4 concentrations and in the absence of TPOAb (1-4). Furthermore, normal TSH weakens the indication for hormone replacement therapy even in the presence of hypothyroxinemia or circulating TPOAb (1-4). Although recent guidelines propose a value of 2.5 mIU/L as the upper limit for TSH in the first trimester of gestation (1-4), they recognize that population variations are possible and that it is important to obtain specific values. Hence, the importance of studies like this.

Our results showed a 97.5^th^ percentile of TSH of 2.7 mIU/L and < 5% of pregnant women with TSH > 2.5 mIU/L. In contrast, also for the Brazilian population, another smaller series (n = 127) found higher TSH concentrations at the end of the first trimester, with a 97.5^th^ percentile of 4.43 mIU/L and 11% of pregnant women with TSH > 2.5 mIU/L (18). Indeed, the upper limit of normal for TSH in the first trimester was > 2.5 mIU/L in many studies, with the 95^th^ percentile reaching 5 mIU/L (1,19,20). One may thus conclude that TSH concentrations < 2.5 mIU/L at the beginning of pregnancy are normal, while concentrations > 5 mIU/L would be clearly altered. The doubt remains whether TSH between 2.5 and 5 mIU/L, especially in the absence of TPOAb and hypothyroxinemia, necessarily indicates thyroid dysfunction. Negro and cols. (21) compared TPOAb-negative pregnant women with TSH < 2.5 mIU/L and between 2.5-5 mIU/L at the beginning of pregnancy and found a higher rate of fetal loss in the latter. No difference in the rate of prematurity was observed in that study (21). Furthermore, it is unclear whether L-T4 replacement therapy in patients with TSH between 2.5 and 5 mIU/L would normalize a possible increased risk. Despite the small sample, we found no additional obstetric or neonatal outcomes in TPOAb-negative women with TSH between 2.5 and 4 mIU/L *versus* TSH < 2.5 mIU/L. It has also been suggested that, within the first trimester of gestation, TSH concentrations until week 6 would be the same as those observed in non-pregnant women and would decrease only after week 7 (20).

In conclusion, our results suggest an upper limit of 2.7 mIU/L for TSH in the first trimester of gestation.
